# Self-management of peripherally inserted central catheters after patient discharge via the WeChat smartphone application: A systematic review and meta-analysis

**DOI:** 10.1371/journal.pone.0202326

**Published:** 2018-08-28

**Authors:** Donghua Ma, Kangwen Cheng, Ping Ding, Hongyan Li, Ping Wang

**Affiliations:** 1 Department of Nursing, People’s Hospital of Tongling City, Tongling, Anhui, China; 2 Attending doctor of Department of Gastrointestinal Surgery, People’s Hospital of Tongling City, Tongling, Anhui, China; 3 Department of Nursing, The First Affiliated Hospital, Anhui Medical University, Hefei, Anhui, China; 4 Department of Urology, The First Affiliated Hospital of Anhui Medical University, Hefei, Anhui, China; Nord University, NORWAY

## Abstract

WeChat is a smartphone application that may help patients self-manage peripherally inserted central catheters (PICC), although additional data are needed regarding this topic. This systematic review and meta-analysis aimed to determine whether WeChat helped improve PICC-related complications, self-care ability, PICC maintenance dependency in that the behavior of a patient is in compliance with a doctor’s order or a will, knowledge mastery, and satisfaction among patients with a PICC. The PubMed, Embase, Cochrane Library, China Biology Medicine, China national Knowledge Infrastructure, Wanfang, Wiper, and Baidu Scholar databases were searched to identify related reports that were published up to April 2018. This search revealed 36 reports that were published during 2014–2018, including 2,623 controls and 2,662 patients who used the WeChat application. Relative to the traditional follow-up group, the group that received WeChat follow-up had a lower risk of PICC-related complications (odds ratio [OR]: 0.23, 95% confidence interval [CI]: 0.19–0.27, *P* < 0.00001), better self-care ability (mean difference: 36.41, 95% CI: 34.68–38.14, *P* < 0.00001), higher PICC maintenance dependency (OR: 4.27, 95% CI: 3.35–5.44, *P* < 0.00001), and higher patient satisfaction (OR: 6.20, 95% CI: 4.32–8.90, *P* < 0.00001). Eight studies reported knowledge mastery, although the different evaluation tools precluded a meta-analysis. Nevertheless, those eight studies revealed that knowledge mastery was significantly higher in the WeChat group than in the traditional follow-up group (*P* < 0.05). To the best of our knowledge, this is the first meta-analysis to evaluate the effects of WeChat follow-up on self-management among patients who are discharged with a PICC. It appears that WeChat follow-up can help improve the incidence of complications, self-care ability, PICC maintenance dependence, and patient satisfaction. However, the WeChat application itself cannot improve patients’ self-management ability. Further studies are needed to produce high-quality evidence to determine whether WeChat is an effective follow-up tool.

## Introduction

Peripherally inserted central catheters (PICCs) have become an alternative to the traditional central venous catheter (CVC). A PICC is usually inserted by a trained nurse who decides to attend and complete special training regarding PICC insertion and management [1]. PICCs are very popular among Chinese patients because of their simplicity, long indwelling time, high level of safety, and low cost [2]. Although patients must regularly maintain the catheter after discharge, most Chinese patients are not able to punctually and correctly maintain their PICC [3], which is related to low education levels, lack awareness regarding the importance of regular PICC maintenance, economic difficulties, and unevenly developed technology and medical conditions in various areas. However, correct PICC maintenance is closely associated with the PICC retention time and complication rate [4], which highlights the importance of continuous nursing to help patients perform regular maintenance of their PICC. The main methods for this nursing involve follow-up via text message, telephone, family visits, and outpatient department visits, although these methods cannot fulfil the increasingly diverse needs of patients and are associated with poor information transmission, high costs, and high rates of loss to follow-up.

The WeChat application was launched in 2011 and has become the most popular social media application among Chinese people, with many researchers exploring its application in the field of continuing nursing [5]. This application offers integrated voice, text, and imaging sharing among a large number of active users, with 549 million monthly active users in 2016 [6]. Relative to traditional medical services, WeChat offers several advantages, including timely ability to contact the patient, a more personalized approach, more convenient operation, and lower costs [7]. This application allows patients to communicate with medical staff, send pictures and video at any time and from anywhere, and receive prompt medical guidance and related information. This approach addresses the space and time limitations of medical services and allows medical staff to provide classified and specific information to patients during their follow-up.

Since 2013, numerous studies have examined the use of WeChat in continuing nursing for PICC cases, although most studies were limited by their small sample sizes and heterogeneous study designs. Furthermore, no systematic review and meta-analysis has examined the effect of WeChat follow-up on the self-management of patients discharged with PICCs. Moreover, no guidelines have been proposed for the self-management of patients discharged with PICCs. Therefore, the present study aimed to evaluate the application of WeChat in this setting, based on clinical research data, to provide evidence for healthcare professionals to make clinical decisions and develop appropriate methods to promote self-management among patients discharged with PICCs.

## Methods

### Search strategy

The PubMed, Embase, Cochrane Library, China Biology Medicine, China National Knowledge Infrastructure, Wanfang, Wiper, and Baidu Scholar databases were systematically and independently searched by DH Ma and KW Cheng. Because WeChat was launched by TenCent Inc. in 2011, the search period was defined as 2011 to April 2018. The search was performed using relevant medical subject headings, keywords, and a Boolean search string, which combined synonyms and MeSH terms(only for the PubMed search, See S1 File.docx) to identify studies that involved a WeChat follow-up and patients who received PICCs. This broad search string was selected to avoid missing studies that might fulfil the inclusion criteria. Reference lists from the identified reports were manually searched to identify any other relevant reports, and the main research groups in this field were contacted to identify any other relevant studies. There were no restrictions regarding language, publication status, or patient race/ethnicity.

### Eligibility and exclusion criteria

Studies were considered eligible if they fulfilled the following criteria: 1) randomized controlled trials (RCTs) that involved a robust randomization method (e.g., random number tables or computer-based randomization), 2) patients who were >18 years old, 3) patients with an indwelling PICC, 4) an intervention time of ≥1 month, 5) an experimental group that received WeChat follow-up and a control group that received traditional follow-up, and 6) studies that evaluated at least one of the primary or secondary outcomes for the present study. The primary outcome was defined as PICC-related complications and the secondary outcomes were defined as self-care ability, PICC maintenance dependency, knowledge mastery, and patient satisfaction. The exclusion criteria were: 1) repeated publication, 2) reports with incomplete data that could not be obtained by contacting the corresponding author, 3) patients with PICC-related complications before the study, and 4) case reports or review articles.

### Study selection process

Two reviewers (DH Ma and KW Cheng) independently assessed the titles and abstracts of the search results to determine their relevance and evaluated the full texts of all potentially eligible studies. Disagreements were resolved via discussion between the two reviewers and involvement of a third reviewer (P Ding) if necessary.

### Data extraction

Two researchers (DH Ma and KW Cheng) independently extracted the data from the included studies, one reviewer checked the data extraction and extracted additional data where necessary, and a fourth reviewer checked all data. The corresponding author was contacted in instances with incomplete or unclear data. Any disagreements regarding the data extraction were resolved via discussion and involvement of a third reviewer (P Ding) if necessary.

The following data were extracted using a pre-prepared data extraction form (See Table 1): basic study characteristics (e.g., first author’s name, year of publication, country, research type), sample size, intervention measures, intervention time, outcome parameters (PICC-related complications, self-care ability, PICC maintenance dependency, knowledge mastery, and patient satisfaction). (See Table 1: Extracted and analyzed data of this systematic literature review.)

**Table 1:** Extracted and analyzed data of this systematic literature review.

Author	Year	Type	Country	Control Group										Experimental Group									
				IT	SS	IM	CP	PS		SA	MDK	MD		IT	SS	IM	CP	PS		SA	MDK	MD	
								S	Dis			De	NDe					S	Dis			De	NDe
ChongyinWei et al [11]	2016	RCT	China	PO	25	C+X	11	17	8					PO	25	Wechat	2	23	2				
Xiaojing jin et al [12]	2017	RCT	China	1M	200	C	34				Scale5			1M	200	Wechat	12				Scale5		
]Huirong Ye et al [13]	2016	RCT	China	1 M	62	C	10					45	17	1 M	85	Wechat+C	7					79	6
Xiaohui Li et al [14]	2016	RCT	China	1 M	60	C					Scale1	42	18	1 M	60	Wechat					Scale1	53	7
Jie Zhao et al [15]	2015	RCT	China	2 M	52	D+O		39	13		Scale2			2 M	68	Wechat+D+O		66	2		Scale2		
Xiaolan Gong et al [16]	2016	RCT	China	2 M	67	O						40	27	2 M	67	Wechat						61	6
Hua Lu et al [17]	2015	RCT	China	2 M	100	O	29							2 M	100	Wechat	6						
WeiWang et al [18]	2014	RCT	China	2 M	100	N		95	5	127.47±9.25				2 M	100	Wechat		99	1	163.32±10.11			
Xuefang Xu et al [19]	2015	RCT	China	3 M	80	O	30					46	34	3 M	80	Wechat	4					65	15
Hongju Fan et al [20]	2016	RCT	China	3 M	30	C	5							3 M	30	Wechat	1						
Xiaoying Wu et al [21]	2016	RCT	China	3 M	100	O+C	38			114.83±9.32	Scale3			3 M	100	Wechat	17			150.61±10.21	Scale3		
Zuyan Fan et al [22]	2016	RCT	China	3 M	38	D	14					29	9	3 M	38	Wechat	2					33	5
Lijie Zhang et al [23]	2016	RCT	China	6 M	90	D	11	82	8			41	49	6 M	90	Wechat	2	89	1			80	10
Caixia Sun et al [24]	2016	RCT	China	6 M	38	C	11							6 M	38	Wechat	4						
Li Zhang et al [25]	2016	RCT	China	12 M	50	O+D		39	11					12 M	50	Wechat+O+D		47	3				
YingyingCheng et al [26]	2018	RCT	ChAina	A··	100	D	34	74	26		NS			3M	100	Wechat	6	94	6		NS		
Rongrong Zheng et al [27]	2017	RCT	China	3M	50	C+D	24			109.32 ± 10.72	Scale3			3M	50	Wechat	9			148.44 ± 10.02	Scale3		
Min Shao et al [28]	2017	RCT	China	3M	70	D	49							3M	70	Wechat	16						
Huiqin Qu et al [29]	2017	RCT	China	12M	115	O	49					65	50	12M	120	Wechat	15					103	17
Liping Lin [30]	2017	RCT	China	6M	52	C		39	13		NS			6M	52	Wechat		50	2		NS		
Lingli Hu et al [31]	2017	RCT	China	3M	150	D	46					78	72	3M	150	Wechat	9					115	35
Lihua Cao et al [32]	2017	RCT	China	4M	30	C	10	21	9			21	9	4M	30	Wechat	3	29	1			28	2
Caiying Meng et al [33]	2017	RCT	China	6M	30	D	12	19	11					6M	30	Wechat	5	28	2				
Shufang Ruan et al [34]	2017	RCT	China	4M	56	O	9							4M	56	Wechat	2						
Qiaoli Xu et al [35]	2017	RCT	China	3M	46	O	13	29	17			17	29	3M	46	Wechat	5	43	3			33	13
Lingmei Zhang [36]	2017	RCT	China	3M	35	O	9							3M	35	Wechat	2						
Qinglan Zhao et al [37]	2015	RCT	China	PO	190	D	25							PO	198	Wechat+QQ	18						
Jinye He et al [38]	2016	RCT	China	PO	55	O	19							PO	55	Wechat	8						
Xiuyan Huang et al [39]	2016	RCT	China	PO	50	D	30							PO	50	Wechat	9						
Li Wang et al [40]	2016	RCT	China	PO	65	C	23	64	1					PO	61	Wechat	6	61	0				
Qin Deng et al [41]	2016	RCT	China	PO	50	C+X	21	45	5					PO	50	Wechat	5	48	2				
SongfengWang et al [42]	2015	RCT	China	PO	100	C+X	19	79	21					PO	102	Wechat	8	94	8				
Caijuan Huang et al [43]	2016	RCT	China	PO	88	C	20	66	22			65	23	PO	76	Wechat	6	76	0			71	5
Li Li et al [44]	2017	RCT	China	PO	100	D	47				Scale4			PO	100	Wechat	17				Scale4		
Huanxia Zhao et al [45]	2017	RCT	China	PO	50	C	13	39	11					PO	51	Wechat	5	48	3				
Fengqing Zhang et al [46]	2018	RCT	China	PO	49	D		39	8					PO	49	Wechat		47	2				

**Abbreviations: IT,** Intervention Time; **SS,** Sample Size; **IM**, Intervention method; **CP**, Complications of PICC; **PS**, Patient Satisfaction; **SA**, Self-care Ability; **MDK**, Mastery degree of PICC knowledge; **MD**, Maintenance Dependency; **S,** satisfied; **Dis**, Dissatisfied; **De**, dependent; **NDe**, Not dependent; **RCT**, randomized controlled trials; **PO**, until the pipe is pulled out; **NS**, no statement; M, month; **C**, discharge instruction; **D**, telephone follow-up; **O**, outpatient department follow-up; **N**, after the hospital does not give any measures; **X,** giving the PICC brochure after discharge; **Scale1:** self-compiled PICC related knowledge questionnaire, including PICC basic knowledge, activities, movement and Observation of complications, a total of 4 dimensions, 25 entries. **Scale 2:** self-compiled scale includes dressing, shower, activities and complications prevention etc. **Scale3:** self-compiled PICC related knowledge questionnaire includes the necessity of PICC implanta‘‘tion, the main points of observation and attention, a total of 3 dimensions,17 entries. **Scale4:** self-compiled PICC related knowledge questionnaire, including the main points of daily life protection, prevention of complications, emergency management of complications, a total of 30 entries. **Scale5:** self-compiled PICC related knowledge questionnaire, the contents include routine maintenance of PICC, self-observation, abnormal situation management, catheter protection, prevention of complications, a total of 10 entries

### Risk of bias assessment

The quality of the included studies was independently assessed by two researchers (HY Li and P Wang) using Cochrane’s risk of bias tool [8]. Table 2 shows the results of the risk of bias assessment based on random sequence generation, allocation concealment, blinding of participants and personnel, blinding of outcome assessment, incomplete outcome data, selective reporting, and other bias. Each evaluation criterion has three options (low, unclear, and high risks of bias) and the report’s quality is subsequently graded as A, B, or C. (Table 2: Risk bias assessment table for each included studies)

**Table 2:** Risk bias assessment table for each included studies.

Author	random sequence generation	allocation concealment	blinding of participants and personnel	blinding of outcome assessment	incomplete outcome data	selective reporting	other bias	quality grade
ChongyinWei 2016	Yes	Unclear	Unclear	Unclear	Yes	Yes	Yes	B
Qinglan Zhao 2015	No	Unclear	Unclear	Unclear	Yes	Yes	Yes	C
Huirong Ye 2016	Yes	Unclear	Unclear	Unclear	Yes	Yes	Yes	B
Xiaohui Li 2016	No	Unclear	Unclear	Unclear	Yes	Yes	Yes	C
Jie Zhao 2015	Yes	Unclear	Unclear	Unclear	Yes	Yes	Yes	B
Xiaolan Gong 2015	Yes	Unclear	Unclear	Unclear	Yes	Yes	Yes	B
Hua Lu 2015	Yes	Unclear	Unclear	Unclear	Yes	Yes	Yes	B
WeiWang 2014	Yes	Unclear	Unclear	Unclear	Yes	Yes	Yes	B
Xuefang Xu 2015	Yes	Unclear	Unclear	Unclear	Yes	Yes	Yes	B
Hongju Fan 2016	Yes	Unclear	Unclear	Unclear	Yes	Yes	Yes	B
Xiaoying Wu 2016	Yes	Unclear	Unclear	Unclear	Yes	Yes	Yes	B
Zuyan Fan 2016	Yes	Unclear	Unclear	Unclear	Yes	Yes	Yes	B
Lijie Zhang 2016	Yes	Unclear	Unclear	Unclear	Yes	Yes	Yes	B
Caixia Sun 2016	Yes	Unclear	Unclear	Unclear	Yes	Yes	Yes	B
Li Zhang 2016	Yes	Unclear	Unclear	Unclear	Yes	Yes	Yes	B
Jinye He 2016	No	Unclear	Unclear	Unclear	Yes	Yes	Yes	C
Xiuyan Huang 2016	Yes	Unclear	Unclear	Unclear	Yes	Yes	Yes	B
Li Wang 2016	Yes	Unclear	Unclear	Unclear	Yes	Yes	Yes	B
Qin Deng 2016	Yes	Unclear	Unclear	Unclear	Yes	Yes	Yes	B
SongfengWang 2015	Yes	Unclear	Unclear	Unclear	Yes	Yes	Yes	B
Caijuan Huang 2016	Yes	Unclear	Unclear	Unclear	Yes	Yes	Yes	B
YingyingCheng 2018	Yes	Unclear	No	Yes	Yes	Yes	Yes	C
Fengqing Zhang 2018	Yes	Unclear	Unclear	Unclear	Yes	Yes	Yes	B
Rongrong Zheng 2017	Yes	Unclear	Unclear	Unclear	Yes	Yes	Yes	B
Min Shao 2017	Yes	Unclear	Unclear	Unclear	Yes	Yes	Yes	B
Huanxia Zhao 2017	Yes	Unclear	Unclear	Unclear	Yes	Yes	Yes	B
Huiqin Qu 2017	Yes	Unclear	Unclear	Unclear	Yes	Yes	Yes	B
Li Li 2017	Yes	Unclear	Unclear	Unclear	Yes	Yes	Yes	B
Liping Lin 2017	Yes	Unclear	Unclear	Unclear	Yes	Yes	Yes	B
Xiaojing jin 2017	Yes	Unclear	Unclear	Unclear	Yes	Yes	Yes	B
Lingli Hu 2017	Yes	Unclear	Unclear	Unclear	Yes	Yes	Yes	B
Lihua Cao 2017	Yes	Unclear	Unclear	Unclear	Yes	Yes	Yes	B
Caiying Meng 2017	Yes	Unclear	Unclear	Unclear	Yes	Yes	Yes	B
Shufang Ruan 2017	Yes	Unclear	Unclear	Unclear	Yes	Yes	Yes	B
Qiaoli Xu 2017	Yes	Unclear	Unclear	Unclear	Yes	Yes	Yes	B
Lingmei Zhang 2017	Yes	Unclear	Unclear	Unclear	Yes	Yes	Yes	B

**Yes:** low risk of bias; **unclear:** unclear risk of bias; **No:** high risk of bias

### Data analysis and statistical methods

The statistical analysis was performed using Review Manager software (version 5.3.; Copenhagen: The Nordic Cochrane Centre, The Cochrane Collaboration, 2014). A χ^2^-based Q-test was used to evaluate inter-study heterogeneity [9], which was considered statistically significant at *P*-values of <0.1. If there was no significant heterogeneity, a fixed effect model was used to evaluate the point estimates and 95% confidence interval (CIs), and a random effect model was used if significant heterogeneity was detected [10]. Results of tests with dichotomous outcomes were expressed as odds ratios (ORs) and 95% CIs, which were analyzed using the Z-test. Results of tests with continuous outcomes were expressed as the mean difference (MD) or standard mean difference (SMD) and 95% CIs.

## Results

### Search results

**Fig 1:**
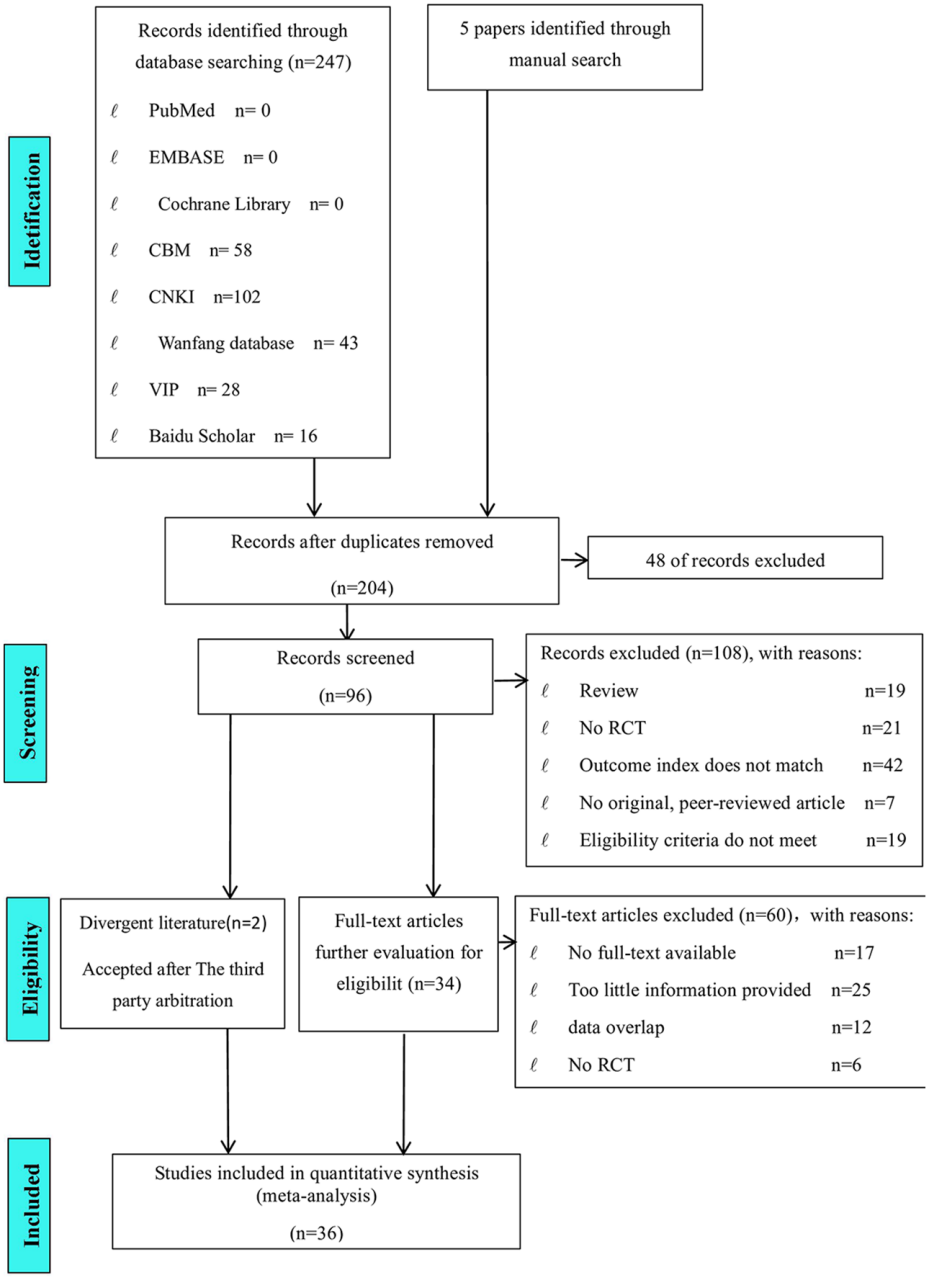
Preferred reporting items for systematic reviews and meta-analyses flowchart depicting the study identification, screening, eligibility and inclusion process.

As shown in Fig 1, **252** reports were initially retrieved using the search strategy, although only **204** reports were retained after removing duplicates. Based on the titles and abstracts, we excluded 108 reports because **19** were reviews, **21** did not describe RCTs, **42** did not include appropriate outcomes, **7** were not original peer-reviewed, and **19** did not fulfil the other eligibility criteria. Based on a review of the full texts, we also excluded **60** reports because the full texts of **17** could not be retrieved, **25** provided too little information, **12** had overlapping data, and **6** did not describe RCTs. Two reports were included after the intervention of the third reviewer because the first two reviewers disagreed on those studies’ inclusion. Therefore, **36** reports were selected for the meta-analysis.

### Study characteristics

The main characteristics of the **36** studies are shown in Table 1. This meta-analysis evaluated **5,285** cases, including **2,662** cases in the WeChat follow-up group and **2,623** cases in the traditional follow-up group. The numbers of cases in each study ranged from **25** [11] to **200** [12]. All study participants had PICCs and were followed-up for **2–12** months, with intervention durations of **1** month in **3** studies [12–14], >1 month in **22** studies [15–36], and until the PICC was removed in **11** studies. Twenty-nine studies [11–13, 17, 19–24, 26–29, 31–45] reported the primary outcome (catheter complications), which included catheter breakage, catheter infection, catheter blockage, thrombosis, and puncture point bleeding. Twenty-eight studies [11–16, 18, 19, 21–23, 25–27, 29–33,35, 36, 40–46] reported the secondary outcomes, including 3 studies [18, 21, 27] that reported self-care ability, **16** studies [11, 15, 18, 23, 25, 26, 30, 32, 33, 35, 40–43, 45, 46] that reported patient satisfaction, **11** studies [13, 14, 16, 19, 22–23, 29, 31, 32, 35,43] that reported PICC maintenance dependency, and **8** studies [12, 14, 15, 21, 26,27, 30, 44] that reported knowledge mastery. Although WeChat was used in the experimental group for all 36 studies, WeChat was combined with other follow-up methods (QQ is an instant messaging software based on Internet developed by Tencent on February 10, 1999. outpatient, or telephone) in **4** studies [13, 15, 25, 37]. (Table 1: Extracted and analyzed data of this systematic literature review.)

### Methodological quality

The quality of the included studies was assessed using the Review Manager quality assessment tool. The random sequence generation (selection bias) was considered low-risk in **33** studies and high-risk in **3** studies. None of the studies involved allocation concealment (selection bias). One study had a high risk based on non-blinding of the participants and personnel (performance bias), while the other studies did not report this parameter. Blinding of outcome assessment (detection bias) was considered low-risk in **1** study, while the other studies did not report this parameter. All studies had low risks of bias based on incomplete outcome data (attrition bias), selective reporting (reporting bias), and other sources of bias (Table 2). Based on those results, **32** studies were assigned a quality grade of B and **4** studies were assigned a quality grade of C.(Table 2: Risk bias assessment table for each included studies)

### Outcome for meta-analysis

#### Effects on PICC-related complications after discharge

**Fig 2:**
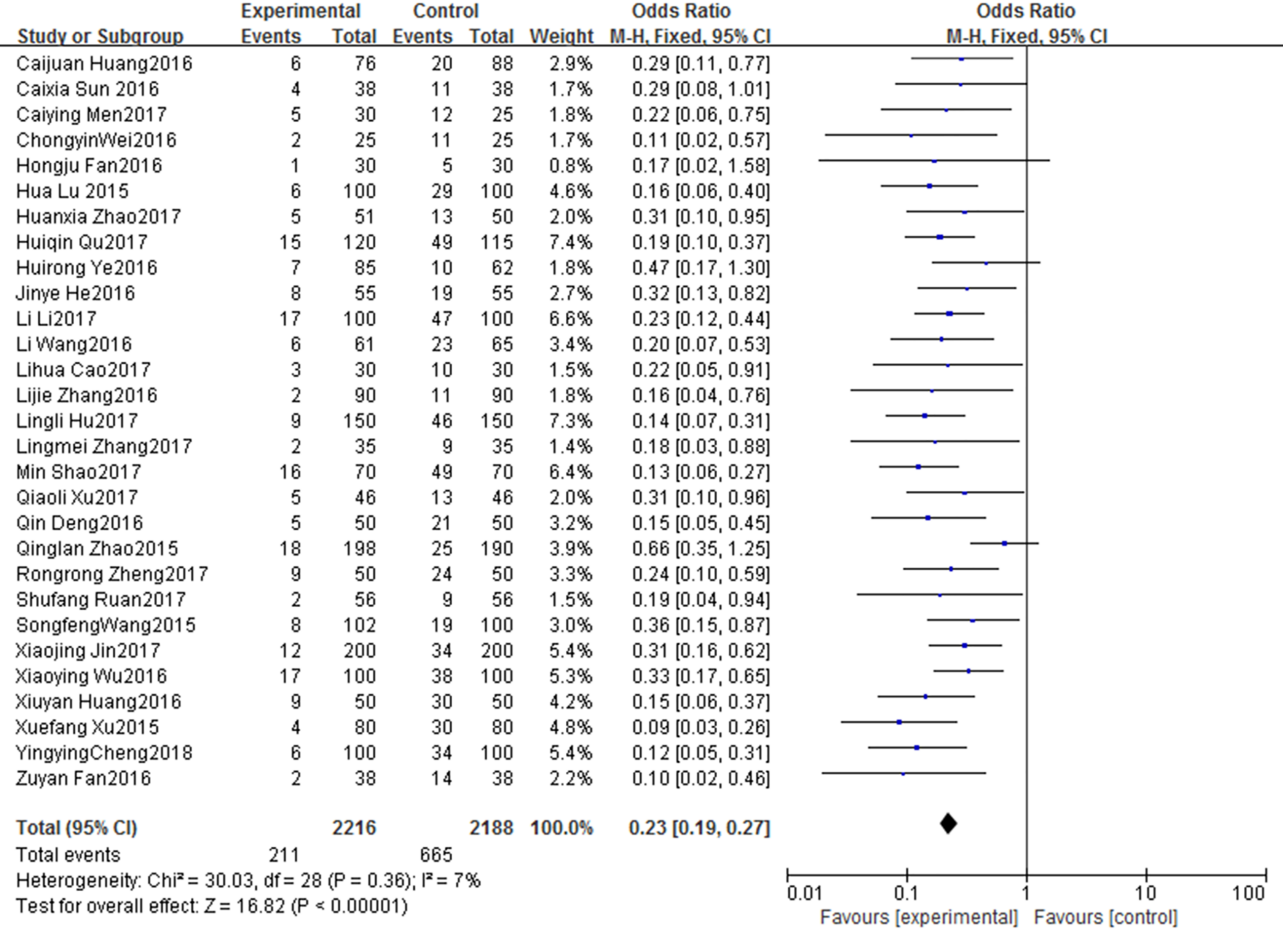
Forest plot diagram showing complications of patients with PICC after discharge in WeChat group and traditional group. Each square indicates a study, and the area of squares is proportional to the weight of the study. The diamond represents the summary OR and 95% CI. CI = confidence interval, OR = odds ratio.

The results of the meta-analysis for PICC-related complications after discharge are shown in Fig 2. Twenty-nine studies [11–13, 17, 19–24, 26–29, 31–45] evaluated this outcome and a fixed effect model was used because there was no significant inter-study heterogeneity (X^2^ = 30.03, df = 28, I^2^ = 7%, *P* = 0.36). The results revealed a significantly lower risk of PICC-related complications in the WeChat group than in the traditional follow-up group (OR: 0.23, 95% CI: 0.19–0.27, *P* < 0.00001) (Fig 2).

#### Effects on patients’ self-care ability

**Fig 3:**
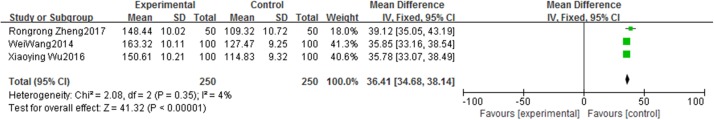
Forest plot diagram showing Self-care ability of patients with PICC after discharge in WeChat group and traditional group. Each square indicates a study, and the area of squares is proportional to the weight of the study. The diamond represents the summary MD and 95% *CI*. *CI* = confidence interval, MD = mean difference.

The results of the meta-analysis for self-care ability after discharge are shown in Fig 3. Three studies [18, 21, 27] evaluated this outcome and a fixed effect model was used because there was no significant inter-study heterogeneity (X^2^ = 2.08, df = 2, I^2^ = 4%, *P* = 0.35). The results revealed significantly better self-care ability in the WeChat group than in the traditional follow-up group (MD: 36.41, 95% CI: 34.68–38.14, *P* < 0. 00001) (Fig 3).

#### Effects on patient satisfaction

**Fig 4:**
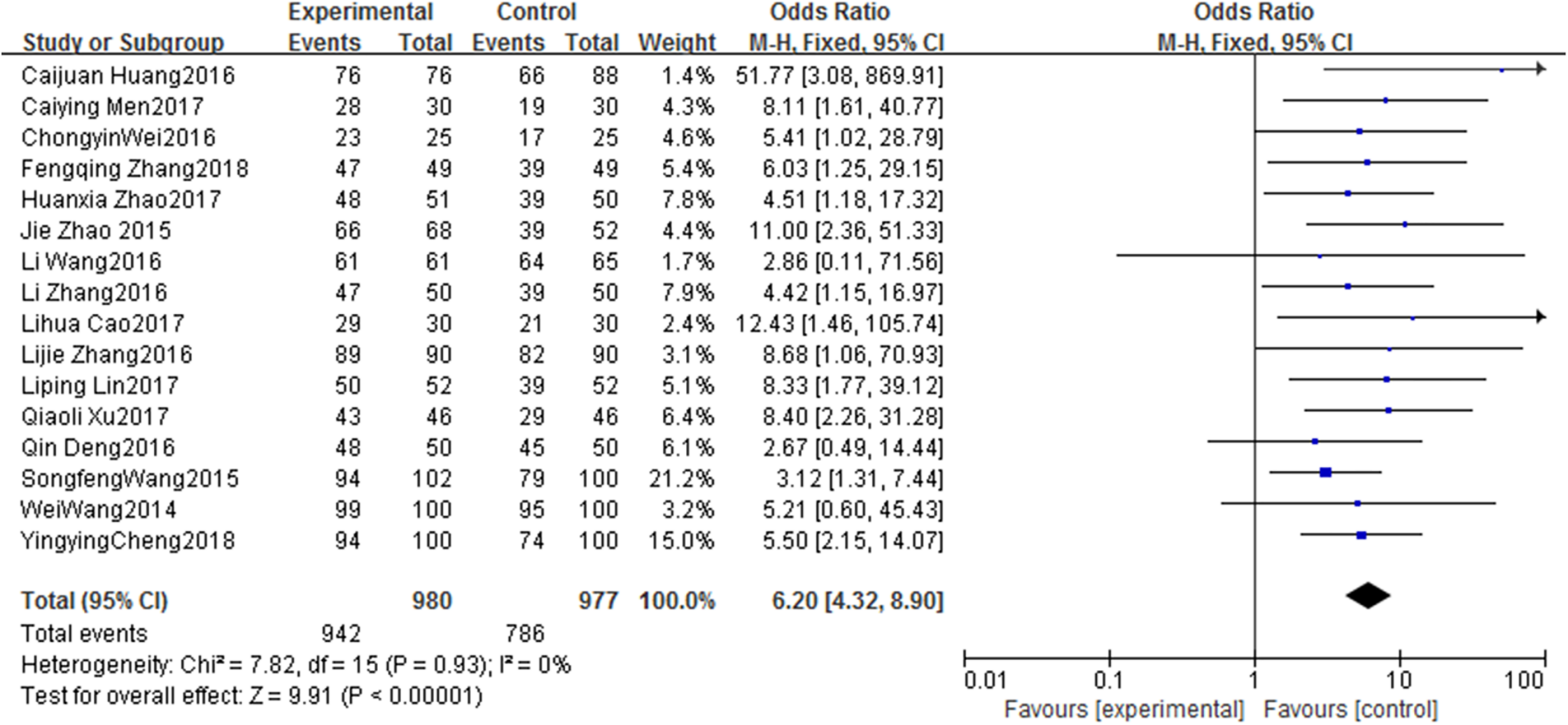
Forest plot diagram showing satisfaction degree of patients with PICC after discharge in WeChat group and traditional group. Each square indicates a study, and the area of squares is proportional to the weight of the study. The diamond represents the summary OR and 95% CI. CI = confidence interval, OR = odds ratio.

The results of the meta-analysis for patient satisfaction are shown in Fig 4. Sixteen studies [11, 15, 18, 23, 25, 26, 30, 32, 33, 35, 40–43, 45, 46] evaluated this outcome and a fixed effect model was used because there was no significant inter-study heterogeneity (X^2^ = 7.82, df = 15, I^2^ = 0%, *P* = 0.93). The results revealed significantly better patient satisfaction in the WeChat group than in the traditional follow-up group (OR: 6.20, 95% CI: 4.32–8.90, *P* < 0.00001) (Fig 4).

#### Effects on PICC maintenance dependency

**Fig 5:**
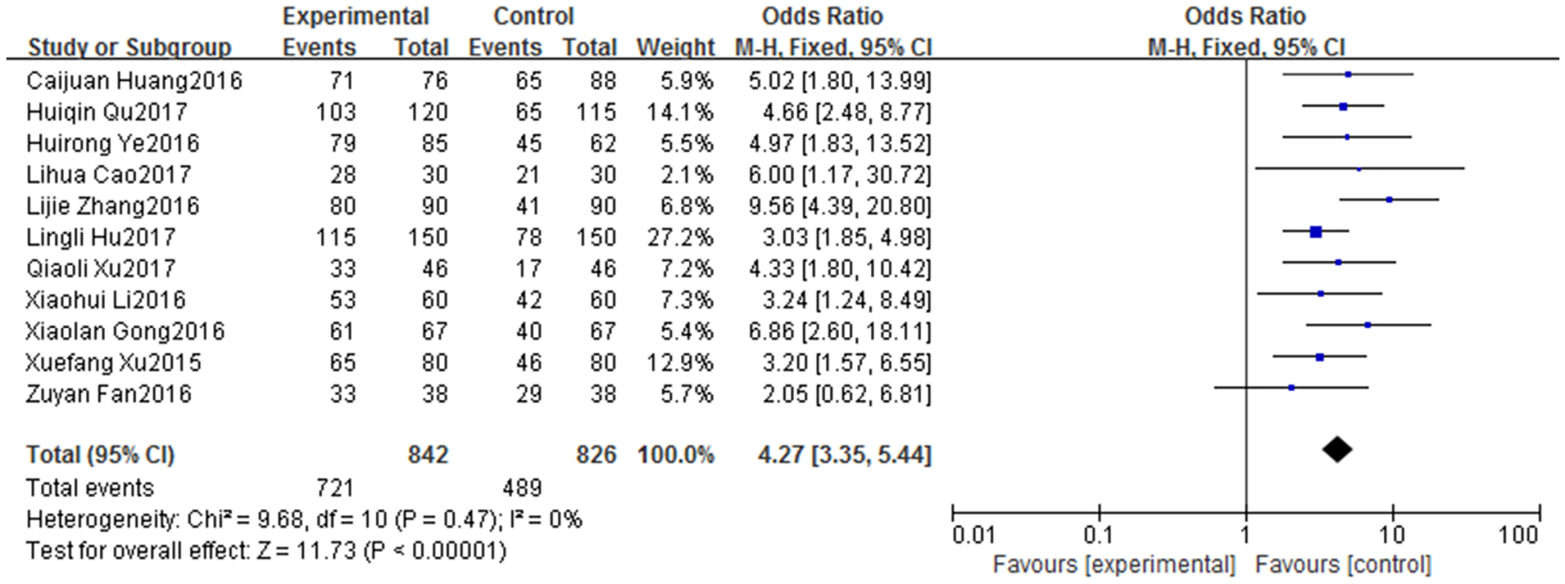
Forest plot diagram showing maintenance dependency of patients with PICC after discharge in WeChat group and traditional group. Each square indicates a study, and the area of squares is proportional to the weight of the study. The diamond represents the summary OR and 95% CI. CI = confidence interval, OR = odds ratio.

The results of the meta-analysis for PICC maintenance dependency are shown in Fig 5. Eleven studies [13, 14, 16, 19, 22, 23, 29, 31, 32, 35, 43] evaluated this outcome and a fixed effect model was used because there was no significant inter-study heterogeneity (X^2^ = 9.68, df = 10, I^2^ = 0%, *P* = 0.47). The results revealed significantly higher dependence on catheter maintenance in the WeChat group than in the traditional follow-up group (OR: 4.27, 95% CI: 3.35–5.44, *P* < 0.00001) (Fig 5).

#### Effects on knowledge mastery

Eight studies [12, 14, 15, 21, 26, 27, 30, 44] evaluated knowledge mastery, although a meta-analysis could not be performed because different tools were used to evaluate this mastery. Li et al. [14] used a self-administered questionnaire with 4 dimensions and 25 items regarding basic PICC knowledge, activities, movement, and observation of complications. Wu et al. [21] and Zheng et al. [27] used a self-administered questionnaire (3 dimensions and 17 items, with higher scores indicating better knowledge mastery) and evaluated the questionnaire’s content validity, which revealed a content validity index (CVI) of 0.922 and a Cronbach’s alpha coefficient of 0.874. Zhao et al. [15] used a self-administered scale regarding various factors, such as dressing, showering, activities, and complication prevention. Cheng et al. [26] and Lin [30] used self-administered questionnaires, although the specific contents were not reported. Li et al. [44] used a self-administered questionnaire that included 30 items that addressed the main points of daily life, prevention of complications, and emergency management. That questionnaire had a CVI of 0.938 and a Cronbach’s alpha coefficient of 0.957. Jin et al. [12] used a self-administered questionnaire that included 10 items that addressed routine maintenance of the PICC, self-observation, abnormal situation management, catheter protection, and prevention of complications. The results of the eight studies revealed significantly greater knowledge mastery in the WeChat experimental groups than in the control groups (*P* < 0.05).

## Discussion

To the best of our knowledge, this is the first meta-analysis to assess self-management among patients discharged with PICCs based on follow-up using traditional methods or WeChat. In the present study, PICC self-management was evaluated based on the patients’ ability to protect the PICC, perform activities, observe and address complications, acquire PICC-related knowledge, and improve catheter maintenance dependency. These factors can help prevent catheter-related complications and improve both catheter and patient safety [47].

### WeChat follow-up can help prevent PICC-related complications

The meta-analysis revealed that, among 4,404 subjects in 29 studies [11–13, 17, 19–24, 26–29, 31–45], WeChat follow-up reduced the risk of PICC-related complications. Cheng et al. [26] reported that WeChat follow-up resulted in an approximately 10% reduction in PICC-related complications relative to telephone follow-up. Similarly, Ruan et al. [34] reported that WeChat follow-up resulted in a 12.5% reduction in PICC-related complications relative to outpatient follow-up. Zhang [36] also reported that WeChat follow-up reduced PICC-related complications by approximately 20% relative to education at discharge, while Zhu et al. [48] reported that WeChat follow-up reduced PICC-related complications by approximately 31% relative to a group that received a PICC education manual. In this context, WeChat follow-up can help reduce the incidence of PICC-related complications because it allows the users to rapidly exchange images, messages, audio recordings, and videos via WeChat groups that allow one or more patients and healthcare professionals to communicate in a timely manner [49]. Furthermore, when healthcare personnel are not immediately available, patients can communicate with each other under the guidance of a specialist [50]. This approach is more convenient and effective than traditional follow-up methods that typically do not allow the patient to rapidly create and upload images [51, 52]. Moreover, WeChat allows patients to receive timely information regarding PICC-related complications, as well as directions to maintain the PICC and avoid or correct these complications.

### WeChat follow-up can improve self-care ability

The meta-analysis revealed that, among 500 subjects in 3 studies [18, 21, 27], WeChat follow-up was better for improving patients’ self-care ability than traditional follow-up. Wu et al. [21] evaluated 200 patients who were discharged with PICCs and followed for 3 months, with the WeChat group having significantly better self-care ability than the control group. Thus, it appears that WeChat-based continuing nursing is more effective than traditional nursing methods in this setting. This is likely because the traditional follow-up methods for PICC maintenance in China cannot meet the needs of patients and their family, and have effects that gradually weaken over time. Moreover, the traditional methods have limited interactions, poor abilities to help the patients learn and comply with the required maintenance, and insufficient personalization, which can all negatively affect patients’ self-care ability. For example, patients who reside in rural settings and have limited education require greater personnel, time, and financial resources to ensure that they maintain their PICC. In addition, most patients and their families perform maintenance at home, but may not be able to read or comprehend the maintenance manual. WeChat follow-up effectively addresses these issues by allowing staff to send videos explaining the PICC maintenance, which can be watched at any time and subsequently allow the patient to improve their self-care ability by repeatedly watching the video. Therefore, this method can allow patients to perform out-of-hospital maintenance, which improves their quality of life and reduces the burden on their families and the healthcare system.

### WeChat follow-up can increase patient satisfaction

The meta-analysis revealed that, among 1,957 subjects in 16 studies [11, 15, 18, 23, 25, 26, 30, 32, 33, 35, 40–43, 45, 46], WeChat follow-up significantly improved patient satisfaction relative to traditional follow-up. Zhang et al. [46] reported that WeChat follow-up provided a 12.3% increase in patient satisfaction among discharged patients with PICCs relative to telephone follow-up. Lin [30] also reported that WeChat follow-up provided an 18.85% increase in patient satisfaction relative to education at discharge, while Xu et al. [35] reported that WeChat provided a 30.43% increase in patient satisfaction relative to outpatient follow-up. These improvements are likely related to the technology behind WeChat allowing patients to receive more dynamic and easily understood guidance, relative to traditional follow-up methods, which allows them to understand and master the required maintenance activities. Furthermore, WeChat allows patients to receive this information at any time and in any place, which improves the effect of the nursing follow-up. Moreover, WeChat allows patients to receive answers from healthcare professionals in real time, and also to send pictures and videos that allow the healthcare professional to better respond to the patient’s query [53].

### WeChat follow-up can increase catheter maintenance dependency

The meta-analysis revealed that, among 1,668 subjects in 11 studies [13, 14, 16, 19, 22, 23, 29, 31, 32, 35, 43], WeChat follow-up significantly improved catheter maintenance dependency by 10–40% relative to traditional follow-up. In this context, the catheter’s behavior directly affects its service life and ability to deliver the desired therapeutic effect [54]. Although Chinese patients with PICCs typically have poor maintenance dependency, traditional follow-up methods do not provide timely and high-quality information transmission to the patient. Moreover, many patients do not receive reimbursement for PICC-related maintenance expenses, which can lead them to believe that delaying maintenance can save them money. In this context, Lyu et al. [55] followed 108 patients with head and neck cancers for 6 months and found that WeChat follow-up was associated with better values for time consumption, economic cost, loss-to-follow-up rate, and patient satisfaction (vs. telephone follow-up). Other studies [19, 23] have also demonstrated that WeChat follow-up improves catheter maintenance dependence relative to telephone follow-up. A specialized WeChat platform allows nurses to send patients maintenance reminders and illustrate the negative effects of not performing maintenance, which will hopefully change the patient’s perception and willingness to perform PICC maintenance.

### WeChat follow-up can improve patients’ knowledge regarding PICC

Eight studies [12, 14, 15, 21, 26, 27, 30, 44]evaluated knowledge mastery using different tools, which precluded a meta-analysis. However, all 8 studies revealed that WeChat was associated with improved knowledge relative to traditional follow-up. This is likely because traditional follow-up relies on the patient passively acquiring knowledge via discharge education, telephone instructions, or an education manual, while most patients actively desire to understand their own disease, treatment methods, rehabilitation methods, and healthcare after discharge. Traditional follow-up methods can partially satisfy these desires, although it is reliant on passive acquisition of knowledge and the efforts of nursing staff [56].

Patients with PICCs have weakened immunity and an impaired self-image as a result of their condition and need for a PICC, which can reduce their willingness to perform public activities. Electronic information dissemination can help address this issue, as smartphones have allowed individuals to seek information at any time and in any place to make full use of their fragmented schedule. Moreover, one study found that 70% of respondents searched for medical information online, including 50.98% of individuals who searched the internet for questions regarding symptom emergence [57]. An American study also revealed that 55% of American adults searched the internet for information regarding health and medical care at least once per month, with 47% reporting that the information influenced their treatment and decision-making [58]. In developed countries, the use of online networks for post-hospital follow-up has become universal, with Wantland et al. [59] reporting that promoting healthy behavior online was more effective than non-network interventions in influencing patients’ knowledge and behavioral changes. However, patients require a professional and reliable platform to ensure that they receive accurate information, and WeChat follow-up platforms in China help address these issues by providing patients with access to professional teams and knowledge, regularly updated information, picture- and video-based explanations, and positive interactions with healthcare professionals. Moreover, Lyu et al. [55] reported that WeChat interventions were more likely to create a friendly relationship between the patient and nurse (vs. telephone interventions), which makes the patient more likely to trust and follow the nurse’s guidance.

### Advantages of WeChat follow-up

WeChat is a free application that was launched in 2011 and had 806 million monthly active Chinese users (>94% of smartphones) in the second quarter of 2016. This application allows the user to rapidly send free voice and text messages, videos, and pictures, as well as share streaming content and notes created using a “voice notepad”. WeChat also features a voice call feature can is very similar to a standard cellphone call. Users can store their electronic media (e.g., text, pictures, videos, and audio) to conveniently and repeatedly view them in the future. These files can be shared one-to-one or with multiple people through a group messaging feature. WeChat is available for most mobile platforms (e.g., iPhone, Android, Windows Mobile, Symbian, and BlackBerry), and is offered in a variety of language interfaces. Although WeChat itself cannot reduce the incidence of PICC-related complications, it can allow healthcare professionals to disseminate information and help patients understand and perform their PICC maintenance. This provides patients with a high level of satisfaction and PICC-related knowledge, which is likely what caused the reduction in PICC-related complications. Finally, WeChat is very simply to operate, even for illiterate users, and is accessible to a broad age range (e.g., 3–70 years old), which allows it to effectively improve the quality of follow-up.

### Insufficiencies of WeChat follow-up

Although WeChat allows nurses to disseminate information, the nurse has no way of knowing whether the patient has viewed that information. Thus, it would be useful to include a feedback function to allow the sender to determine whether the recipient has viewed the message. Second, WeChat relies on access to a mobile network, which may mean that traditional follow-ups are more appropriate when mobile access is not regionally available. Thus, it is possible that a combination of WeChat and traditional follow-up methods may help improve follow-up quality.

### Study limitations

The present study has several methodological limitations. First, the overall quality of the 36 included studies was relatively low, and one outcome (knowledge mastery) could not be evaluated in the meta-analysis because of heterogeneity in the evaluation tools. Therefore, the results of this meta-analysis should be interpreted cautiously, and large high-quality RCTs are needed to confirm our findings. Second, the meta-analysis did not consider the specific WeChat intervention methodology and frequency, which should be evaluated in future studies.

### Summary

In conclusion, this systematic review and meta-analysis revealed that WeChat follow-up improved PICC self-management relative to traditional follow-up methods, with moderate beneficial effects on PICC-related complications. However, further well-designed large prospective studies are needed to validate our findings.

## Supporting information

S1 File PubMed search strategy. (DOCX)

S2 File PRISMA 2009 checklist. (DOC)
