# Supplementary material for: Self-management of peripherally inserted central catheters after patient discharge via the WeChat smartphone application: A systematic review and meta-analysis
Source: PLoS One. 2018 Aug 28;13(8):e0202326. doi: 10.1371/journal.pone.0202326 (PMC6112638; doi:10.1371/journal.pone.0202326)
Supplement: S1 File — (DOCX) [file pone.0202326.s001.docx]

PubMed Search Strategy

Few articles about the application of WeChat in the follow-up of patients with PICC, If there are too many search restrictions may lead to missed detection, therefore, in this study, WeChat and PICC are used as the key words, and the literature after retrieval is manually screened to ensure that the literature retrieval is complete. But it doesn't leak. (all search strategies are the same as PubMed.)

The following is the PubMed search strategy

Peripherally Inserted Central Catheter, "Catheterization, Peripheral"[Mesh], PICC

#1 ((PICC) OR "Catheterization, Peripheral"[Mesh]) OR Peripherally Inserted Central Catheter

WeChat, WeChat application, WeChat platform

#2 ((WeChat) OR WeChat application) OR WeChat platform

#3 #1 AND #2

((((PICC) OR "Catheterization, Peripheral"[Mesh]) OR Peripherally Inserted Central Catheter)) AND (((WeChat platform) OR WeChat application) OR WeChat)
